# Infection prevention and control research priorities: what do we need to combat healthcare-associated infections and antimicrobial resistance? Results of a narrative literature review and survey analysis

**DOI:** 10.1186/s13756-020-00801-x

**Published:** 2020-08-24

**Authors:** Yohann Lacotte, Christine Årdal, Marie-Cécile Ploy, on behalf of the European Union Joint Action on Antimicrobial Resistance and Healthcare-Associated Infections (EU-JAMRAI)

**Affiliations:** 1 University of Limoges, INSERM, CHU Limoges, RESINFIT, U1092, F-87000 Limoges, France; 2 Antimicrobial Resistance Centre, Norwegian Institute of Public Health, Oslo, Norway

**Keywords:** Antimicrobial resistance, Infection prevention and control, Healthcare-associated infections, Research priorities

## Abstract

**Background:**

Infection prevention and control (IPC) is one of the most cost-effective interventions against antimicrobial resistance (AMR). Yet, IPC knowledge gaps often receive little prominence in AMR research agendas. In this article, we construct IPC research priorities, in order to draw attention to these critical research needs.

**Methods:**

We developed a 4-step framework to identify IPC knowledge gaps from literature (narrative review). These gaps were then translated into research priorities and sent to two groups of European IPC experts for validation and critique through an online survey.

**Results:**

Seventy-nine publications were retrieved from the literature review, identifying fifteen IPC research gaps. Forty-four IPC experts, clustered in two groups, vetted them. The experts classified all research gaps as medium or high priority. Overall agreement between both groups was average (Kendall’s τ = 0.43), with strong alignment on the highest priorities: (i) the assessment of organizational, socio-economic, and behavioural barriers/facilitators for the implementation of IPC programmes, (ii) the impact of overcrowding on the spread of infections and (iii) the impact of infrastructural changes, at facility level, on the reduction of infections. Feedback from experts also identified an additional research gap on the interaction between the human and hospital microbiomes.

**Conclusions:**

We formulated a list of sixteen research priorities and identified three urgent needs. Now, we encourage researchers, funding agencies, policymakers and relevant stakeholders to start addressing the identified gaps.

## Introduction

Antimicrobial resistance (AMR) is a growing health issue with the potential to undermine modern medicine. In 2015, 670,000 infections with antibiotic-resistant bacteria were reported in Europe accounting for 33,000 deaths [1]. If AMR rates continue to increase and follow the predicted trend, 2.4 million people could die from resistant bacteria in Europe, North America and Australia between 2015 and 2050 [2]. Bacteria will always evolve resistance to antibiotics. Yet, this evolution can be hindered through a broad set of interventions combining surveillance, antibiotic stewardship, infection prevention and control (IPC) and ensuring appropriate access to antibiotics while maintaining efforts to bring new innovative antibiotics (or alternative therapeutics), diagnostics, and vaccines to the market [3]. All these interventions need to be applied in a One Health perspective, considering the interaction between humans, animals and the environment [4].

Each intervention area comes with knowledge gaps. It is important that these gaps are aggregated and communicated as research priorities so that both national and international actors may concert their efforts on critical needs, avoiding duplication and ensuring that the resulting evidence informs policies [5]. For the past few years, several initiatives have compiled strategic research agendas covering AMR. Among them, the Joint Programming initiative on Antimicrobial Resistance (JPIAMR, www.jpiamr.eu/) Strategic Research and Innovation Agenda (SRIA), launched in 2014 and updated in 2019, covers the full breadth of AMR research in a One Health context [6]. In 2014, EU’s Innovative Medicines Initiative (IMI, https://www.imi.europa.eu/) published a strategic research agenda compiling the innovation priorities for new medicines and other technologies across a range of therapeutic areas, including AMR [7]. Finally, in 2019, the One Health European Joint Programme (One Health-EJP, www.onehealthejp.eu/) published a strategic research agenda in the area of foodborne zoonoses and prevention of transmission of AMR in the food chain [8].

To assess the uptake of these agendas within Europe, the European Joint Action on Antimicrobial Resistance and Healthcare-Associated Infections (EU-JAMRAI, www.eu-jamrai.eu/) previously compared the AMR-related research priorities of seven participating countries with the comprehensive JPIAMR strategic research agenda [9]. This comparison revealed three potential gaps: (i) clinical trials efficiency, (ii) AMR in the food chain and (iii) IPC. Yet, the first two gaps are respectively covered by the IMI agenda (partially) and the One Health-EJP agenda, meaning that clear research directions are available for policymakers and funders. Regarding IPC, the JPIAMR SRIA outlines six research priorities of which three address IPC on a general level. We therefore believe that there is a gap to fill here and that specific IPC research priorities, validated by IPC experts, are a valuable addition to the existing multi-country strategic agendas.

Indeed, IPC can be one of the most cost-effective interventions to combat AMR. A recent report from the Organisation for Economic Co-operation and Development (OECD) estimated that promoting simple IPC measures such as hand hygiene could reduce by about 40% the AMR health burden [2]. Improving IPC would also help to reduce the multitude of non-resistant healthcare-associated infections (HCAI) causing millions of extra days in hospital [10], and representing a financial loss of several billion euros each year [11]. Despite their tremendous importance, many IPC measures are still based upon insufficient evidence (as assessed through the GRADE methodology), e.g. guidelines for preventing surgical site infections [12]. Additionally, there is a paucity of studies evaluating the cost-effectiveness of IPC interventions, which are needed to help countries determine how to best improve IPC. Research in the area is therefore needed and specific research priorities would help engaging and coordinating efforts to tackle urgent needs.

This study provides detailed IPC research priorities, validated and supported by IPC experts, which is a valuable tool for researchers, funding agencies and policymakers to fill knowledge and research gaps.

## Methods

### Narrative literature review and gap identification

**Fig. 1:**
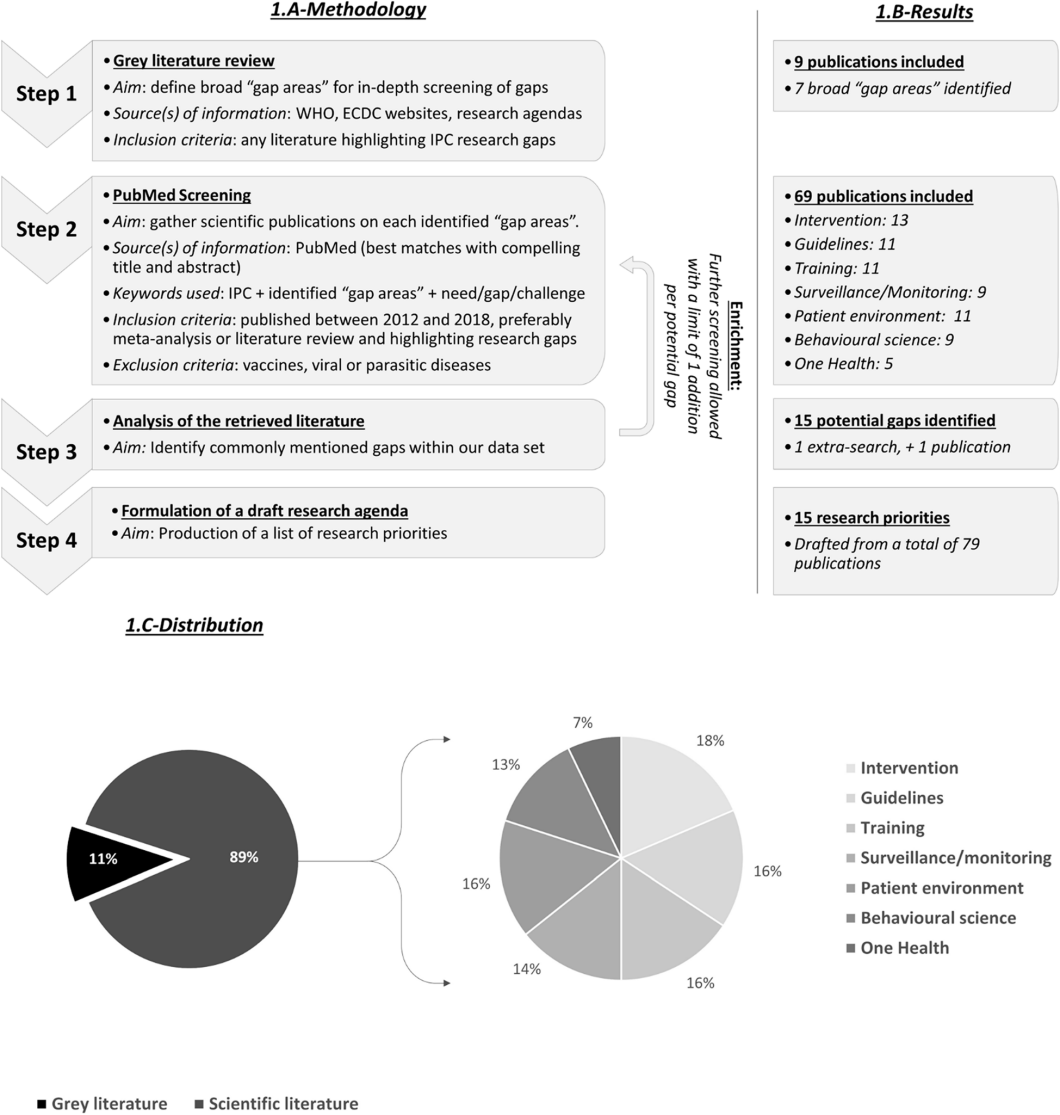
Overview of the 4-step narrative review framework used to build the draft IPC research priorities. **a**-Methodology. A 4-step framework was used to perform the literature review and build the draft research priorities. **b**-Results. Seventy-nine publications were retrieved from the literature review. **c**-Distribution. Overall, publications were fairly distributed between pillars. One Health literature may have been underrepresented

To identify knowledge gaps, we performed a narrative literature review following a 4-step framework (Fig. 1.A). First step consisted in a grey literature review to shape our analysis and identify broad “gap areas” for in-depth screening. Within second step, we screened PubMed for articles, published between 2012 and December 2018, highlighting knowledge gaps on each of the identified gap areas. Keywords used for the screening are mentioned on Fig. 1.A. We selected articles based on their title, abstract and content, favouring meta-analyses or literature reviews. We specifically excluded literature on vaccines since research priorities have already been documented [13, 14], and literature on viral/parasitic diseases since our focus here is bacterial resistance. Thirdly, we analysed the literature, determining commonalities between articles to identify important knowledge gaps. When relevant gaps were raised but without enough information in our dataset, additional PubMed searches were performed to gain understanding (enrichment process). Finally, we formulated a draft of research priorities based on all information retrieved from previous steps.

### Survey and gap validation

To validate the aggregated research priorities and identify the most pressing needs, our draft research priorities were sent to European IPC experts for review through an online survey.

The survey was composed of three questions (Additional file 1). The first question assessed the urgency of each identified research priority. Six answers were available: (i) not a priority, (ii) low priority, (iii) medium priority, (iv) high priority, (v) critical priority and (vi) I don’t know. The second question asked for comments on each research priority, including suggestions for modifications of research priority. The third question was an open comment section where additional research priorities could be added.

The survey was sent to two groups of experts to assess inter-agreement. The first target group was composed of 18 IPC experts from 11 European countries. Experts from this group were selected on their publication record and/or involvement in specific organizations, making sure to have enough diversity (eleven European countries, one European organisation and one One-Health organisation). The second target group was composed of 33 members from the European Committee on Infection Control group (EUCIC, https://www.escmid.org/eucic/), a sub-group from the European Society for Clinical Microbiology and Infectious Diseases (ESCMID). The survey was distributed in February/March 2019 to the first group and in September 2019 to the second, upon agreement with the EUCIC Executive Board. There was no overlap between both groups.

### Analysis

Answers from first question were scored a value of 0 to 4 (0 corresponding to “not a priority” and 4 to “critical priority”). Based on this scoring system and answers from the survey, we calculated the average priority of each of the identified research needs and ranked them according to their priority level. This was done for both target groups and for merged groups. To assess inter-agreement between both groups and check whether they agree on the most urgent needs, we calculated (i) the linearly weighted Cohen’s kappa coefficient to measure agreement on classification (either no, low, medium, high or critical priority) and (ii) the Kendall rank correlation coefficient to measure agreement on ranking.

For critical analysis, comments from the second question were labelled as either (i) supportive, (ii) informative (when adding insight on a topic), (iii) critical or (iv) calling for modifications. Comments from the third question were reviewed individually to identify potentially missed research needs. Revision of the draft research priorities was considered when a modification or addition was requested by more than 5% of the total respondent population with requests coming from both groups.

## Results

Results of our 4-step review are available in Fig. 1.B. We identified nine publications from grey literature in step 1: four reports from the European Centre for Disease Prevention and Control (ECDC), two from the World Health Organisation (WHO) and three international research agendas (JPIAMR, IMI and One Health-EJP’s). They allowed to define seven broad “gap areas” requiring in-depth screening: (i) IPC interventions, (ii) guidelines, (iii) training, (iv) surveillance/monitoring, (v) patient environment (facilities and staffing), (vi) behavioural science, and (vii) One Health. In step 2, we screened Pubmed and retrieved 69 relevant articles. Stratification of these 69 articles between gap areas is available in Fig. 1.B. Enrichment process led to the inclusion of one additional publication on syndromic-based surveillance (surveillance based on available patient clinical information rather than microbiological data for early detection of infection), for a total of 70 publications evenly distributed across gap areas (Fig. 1.C). Based upon grey and scientific literature, we proposed a draft list of 15 research priorities clustered in 7 categories (Table 1, column 1).

**Table 1:** Infection Prevention and Control Research Priorities (including survey vetting scores)

Research priorities	***Weighted average criticality***	***Priority category***	***Priority ranking***
**IPC interventions**			
1. There is a lack of high-quality studies addressing the effectiveness of hospital-based IPC programmes, including their impact, cost-effectiveness, and ideal composition.	3.20 *	High	4
	2.87	Medium	
	2.95 *	Medium	
**IPC guidelines**			
2. Many best practice IPC recommendations are based upon weak evidence. For example, the World Health Organization identified, in its Global Guidelines for the Prevention of Surgical Site Infection, 20 recommendations with a “low” quality of evidence. The evidence base supporting IPC guidelines needs to be strengthened.	2.82	Medium	6
	2.91 *	Medium	
	2.89	Medium	
3. Situational analyses in different settings (high, medium or low-incomes countries) but also different healthcare settings (intensive care units, short or long stay, medico-social facilities) are needed to better understand potential adaptations of IPC guidelines.	2.45	Medium	7
	2.91 *	Medium	
	2.79	Medium	
4. A better understanding of the different patient screening strategies is needed for risk management. This includes who should be screened, when (including start and stop of screening), and how movement between healthcare institutions should trigger screening. Research should include both clinical impact and cost-effectiveness.	2.50	Medium	9
	2.78	Medium	
	2.71	Medium	
**IPC training**			
5. Additional tools are needed to evaluate IPC training programmes and implement them.	2.82	Medium	14
	2.44	Medium	
	2.53	Medium	
6. New innovative ways of training should be evaluated such as e-learning, simulation, self-directed training modules or mentorship for IPC education. There is a lack of study on the impact of these innovative training tools on the practice change and infection rate in healthcare facilities.	2.91	Medium	8
	2.66	Medium	
	2.72	Medium	
7. Minimal standard requirements for the recruitment and training of IPC professionals should be investigated.	2.30	Medium	13
	2.63	Medium	
	2.55	Medium	
**IPC surveillance and monitoring**			
8. Research is needed to assess and validate the reliability of surveillance based on available patient clinical information (syndromic-based surveillance) rather than microbiological data or prescription databases, i.e., data gathered for other primary purposes.	1.90	Low	11
	2.78	Medium	
	2.57	Medium	
9. There is a lack of published standards to monitor IPC practices beyond hand hygiene. Evidence-based standardised audit protocols need to be created addressing, for example, catheter-related bloodstream/urinary tract infections and ventilator-associated pneumonia.	3.09 *	High	5
	2.84	Medium	
	2.91 *	Medium	
10. There are a number of innovative, new methods to monitor compliance to IPC practices, including electronic and infrared approaches. These need to be tested in multiple settings to assess their value for IPC programmes.	2.73	Medium	15
	2.39	Medium	
	2.48	Medium	
**Impact of patient environment on HCAI and AMR reduction (facilities and staffing)**			
11. Insufficient data are available on the impact of infrastructural changes at the facility level on the reduction of infections and resistance. This includes the accessibility to specific equipment, density of hand washing points, availability of single occupancy rooms, and more.	3.00 *	High	3
	2.94 *	Medium	
	2.95 *	Medium	
12. Research is needed to explore the impact of patient-to-bed ratio on the spread of infections and resistance, including instances of overcrowding. This should include analyses of staff workload, available staffing (including presence of IPC professionals), bed occupancy, and visitor frequency.	3.36 *	High	2
	2.97 *	Medium	
	3.07 *	High	
13. Research is needed to study the interaction between the human and hospital microbiome.**	n/a	n/a	n/a
**Behavioural science**			
14. Studies are needed to assess the demographic, organizational, economic, sociological, and behavioural factors facilitating success but also the barriers and challenges to implement effective IPC programmes.	3.55 *	High	1
	3.00 *	High	
	3.14 *	High	
15. Patients and their families are key elements in the chain of transmission in healthcare facilities. Studies addressing the impact of patient and family-oriented education and communication campaigns (involving patients associations) on the rate of hospital-acquired infections are needed.	2.73	Medium	10
	2.63	Medium	
	2.65	Medium	
**One Health**			
16. Research is needed to assess the impact of IPC measures in different operational contexts including small farms, industrial farms, feedlots, slaughterhouses, fish farms, and more. IPC measures may include the density of the animal populations, vaccination, hygiene measures and antibiotic use.	2.60	Medium	12
	2.56	Medium	
	2.57	Medium	

Through our literature review, we extracted a list of 15 IPC research priorities. They are presented in the first column of this table. Each of them was surveyed by two groups of IPC experts. Experts were asked how urgent each of the identified gap was. Answers were scored a value of 0 to 4 (0 corresponding to “not a priority” and 4 to “critical priority”). Based on this scoring and results from the survey, we calculated the weighted average criticality of each assumption (second column) and assigned them into a priority category (third column). For each assumption, three results are presented. First line corresponds to the results obtained with the first target-group composed of 18 European IPC experts. Second line correspond to the results obtained with the second target-group, EUCIC members. Third line correspond to merged results from both groups. In each group, the top five research needs, according to experts, are highlighted by a * mark. Finally, research priorities were ranked from 1 to 15, from the most to the less urgent one, based on merged results from both groups. Results of this ranking are presented in the fourth column. The survey also allowed to identify an additional research priority. It is highlighted by a ** mark in the table. For this additional priority, no weighted average criticality, priority category nor ranking was calculated as it was not included in the survey

This draft list was then vetted by two expert groups through an identical online survey. With the first group, composed of 18 selected European IPC experts, we achieved a response rate of 61% (11/18 respondents). For the second group, targeting the EUCIC members, we gathered 33 answers through a two-week open consultation on the EUCIC website.

Overall, there was strong support for the draft research priorities. All priorities were found on average to be of medium priority or higher (Table 1, merged groups). Within the first target group, five research gaps emerged as high priority topics, nine as medium priority topics and only one as a low priority topic. In the second target group, only one research gap was considered as a high priority topic while all others ranked in the medium priority category. Overall agreement between both groups on their classification of research gaps appeared fair with a Cohen’s κ = 0.21. When looking at priority ranking, concordance between both groups is better with a 42.9% agreement as assessed by the Kendall rank correlation coefficient (z-score = 2.23, *p*-value = 0.026), indicating an overall average level of agreement. However, a strong alignment on the two most urgent needs was apparent when looking at both group results (Table 1). These critical gaps, 14 and 12, concern respectively (i) the assessment of the demographic, organizational, socio-economic and behavioural barriers/facilitators for the implementation IPC programmes, and (ii) the impact of overcrowding on the spread of HCAI and AMR. Additionally, priority 11 (impact of infrastructural changes at facility level on the reduction of HCAI and AMR) was also in the top five of both priority rankings (Table 1, * mark).

We received 48 comments on our draft priorities. They included 17 comments considered as supportive, 22 as informative, five as critical, and four suggested modifications or rephrasing. All modifications were requested by less than 5% of the experts and thus were not included in the final list of priorities. Regarding critics, some experts (7%) questioned the need for additional IPC evidence. They argued that “high quality” evidence is hard to produce in the field due to methodological concerns and that evidence, while not “high quality”, may already be numerous enough. Other experts (5%) expressed that the highest priority is implementation rather than research.

Fourteen additional research priorities were proposed by experts but only one was mentioned enough to be included in the final list: “research on the interaction between the patient and hospital microbiome” which was mentioned by 9% of the experts and by both groups. According to this feedback, we proposed the final 16 research priorities mentioned in Table 1.

## Discussion

The aim of this study was to generate IPC research priorities that could be used by policymakers, funders, and researchers to elucidate important IPC knowledge gaps. We constructed this list using an approach combining (i) a 4-step narrative literature review to identify knowledge gaps and (ii) a validation process with the help of two groups of IPC experts responding to a survey.

Survey results clearly demonstrated the need for research on IPC since several research gaps were scored as high priority areas in both target-groups. Results also support our attempt at building a list of important priorities with (i) no research gap bellow the medium priority (merged groups), (ii) mostly supportive and informative comments on research gaps, (iii) only few requests for additions. According to feedbacks, we only made one additional priority on the interaction between the human and hospital microbiomes (Table 1, ** mark). It was deemed a suitable addition since requested by both expert groups and by 9.1% of the whole expert population. Furthermore, several studies have shown the role of the microbiota in preventing acquisition or expansion of HCAI [15, 16], but with studies limited to murine models or clinical studies with small numbers of patients [17].

Through our analysis, three research gaps emerged as particularly important from both expert groups.

### Assessment of demographic, organisational, socio-economic, and behavioural barriers/facilitators to implement effective IPC programmes

Over the past few years, socio-economic and behavioural sciences have greatly contributed to the fight against HCAI by identifying barriers and facilitators for the implementation of IPC measures. Commonly mentioned barriers include a lack of training/knowledge or awareness [18, 19], and a lack of institutional resources, especially in low- and middle-income countries (LMIC) [19, 20]. On the contrary, close relationships between healthcare workers [21], positive leadership and role modelling are often seen as facilitating factors [22, 23]. While behavioural determinants have been identified, only few interventions have been proposed and tested to address them. An impactful intervention could be, for instance, the appointment of an IPC champion in institutions to help engage and educate colleagues [24]. Yet, limited quality of evidence failed to generate concrete recommendations. More research is therefore needed to assess innovative interventions and to test organisational frameworks that facilitate the implementation of IPC measures. Interestingly, this research gap is mentioned in the JPIAMR SRIA [6]. The JPIAMR could therefore help funding research in the area.

### The impact of overcrowding (staff workload/availability, bed occupancy, …) on the spread of HCAI

There is growing evidence on the impact of overcrowding on HCAI transmission rate. Low staffing and increased workload have been associated with a higher risk of HCAI acquisition [25, 26]. Regarding bed occupancy, the literature is still inconsistent [27–29]. These discrepancies could be explained by differences in study settings, monitoring outcomes but mostly by differences in methodologies and bed occupancy definition. More research is therefore needed but with appropriate occupancy parametrization [30]. Overall, there is still insufficient data to generate clear and robust breakpoint thresholds needed by policymakers and hospital managers to implement effective actions (worker/patient and patient/bed ratio for instance). Ideally, breakpoints should be defined for various healthcare settings (intensive care units, short or long stay wards, long-term care facilities) and country settings (high, medium, and low-resource settings). More studies on the impact of visitor frequency and patient movements on HCAI transmission rates would also be beneficial to explore new interventions.

### Assessment of the impact of infrastructural changes at facility level on the reduction of infections and resistance

There are little data available on the impact of infrastructural changes on HCAI. In 2016, a meta-analysis concluded that a high density of hand-washing points and single-patient rooms could help reducing HCAI transmission rates in short-term care facilities [31]. However, these conclusions present some major limitations: (i) the small number of studies included in the meta-analysis, (ii) several studies were uncontrolled before and after intervention and (iii) several studies included in the meta-analysis were biased by bundle effects. More research in the area is therefore needed. However, as highlighted by experts, infrastructural changes are rarely considered as research opportunities. Ideally, IPC outcomes should be studied for any new healthcare facility or any facility remodelling. For instance, purchase of sinks, showers or bathtubs in healthcare institutions should include an analysis of evidence of how easily they can be disinfected. Placement and design of hand sanitisers should be based upon evidence on where healthcare personnel are most likely to use them.

Interestingly, not all IPC experts agreed that there is a need for additional IPC research, despite the dearth of high-quality evidence clearly displayed in many of IPC guidelines [12, 32]. The main thrust of this feedback is that these experts would rather have the funds to implement IPC than to research them. We are sympathetic to this argument, as it is a priority to implement effective IPC measures. However, these funding sources are not necessarily competitive, as implementation funds would normally come for the healthcare budget and research funds from the research budget as well as multinational funding sources like JPIAMR. We believe that effective implementation can run in parallel to ongoing research, as it does in other fields.

Other feedback included the difficulty of producing “high quality” IPC research, as determined through the GRADE methodology, which considers randomized control trials (RCTs) as gold standard. While RCTs, especially clustered randomized trials, are appropriate to evaluate some individual elements of IPC programmes (surveillance for instance), they are often limited when assessing IPC programmes containing multiple interventions or relying on qualitative measurements. For example, RCTs are not suited to evaluate organisational or behavioural interventions which rely on measurements such as governance, commitment or compliance. RCTs may also be inappropriate for IPC interventions due to sample size, ethical limitations or even feasibility. However, GRADE does allow for other types of studies to generate high quality of evidence, like cohort, case-control, before-after and time series studies [33]. These study types would be more suited for the three urgent needs identified and could provide meaningful evidence. For instance, there are new developments in data and analytical technologies, offering the opportunity for observational studies to provide much stronger evidence. Propensity score matching, now, allows the assembly of two or more groups such that they appear to have been randomized to a comparator [34]. Improvement in data collection and data linkage techniques also make observational studies easier to undertake [35]. Powerful observational studies could help to provide evidence on barriers/facilitators for IPC measures implementation, thereby tailoring the design of innovative interventions. Implementation studies could then help testing these interventions. Contrary to local and time limited qualitative studies, implementation studies can match qualitative data with measures of success and process indicators over time, generating high quality evidence [36, 37]. In the end, all of these study types could strengthen meta-analyses and provide gold standard evidence related to IPC**.** In some instances, methodological work is also needed to define appropriate parametrisation or standards to undertake research. This is notably the case for research on the link between bed occupancy and HCAI where different parametrisations of bed occupancy have led to conflicting findings.

There are limitations to our study, specifically the small number of survey respondents (*n* = 44). However, this small number of respondents also reflects our strategy to target known European IPC experts/expert groups, which drastically reduces sample size. The average level of inter-agreement between both target-groups (Cohen’s κ = 0.21 and Kendall’s τ = 0.43) could also be interpreted as a lack of agreement on the most urgent needs. However, Cohen’s κ have been shown to be naturally lower when computing more than three categories (five in our survey) [38]. Inter-agreement on sorting could therefore be underestimated by our statistical test. Regarding inter-agreement on ranking, while the computer Kendall’s τ remains average, we clearly have a strong expert alignment on the most urgent needs with top two research needs being the same in both groups. Also, we are aware that differences in the number of respondents (11 versus 33) may impact agreement between both target groups. Another limitation is that the experts we interviewed were only from high-income countries. Although we did include LMICs in our literature analysis and proposed research priorities targeted toward them, LMICs were excluded from the validation process. A further study, focusing on LMICs, should be conducted to validate all our findings in this setting. Lastly, we had only one broad research gap focused on IPC measures for agriculture, livestock and the environment, given our panel of human health experts. More focused and detailed IPC research priorities on animals and environment would be beneficial.

Despite its limitations, we believe that this study will inform policymakers and funding agencies regarding important IPC research priorities.

## Conclusion

IPC, as demonstrated through the OECD study, can be one of the most cost-effective interventions to guard against AMR. It is also essential to improve overall health outcomes at healthcare institutions. Therefore, it is remarkable that the research needs of IPC have been to date undervalued. IPC often pertains to tasks like handwashing and instrument disinfection, which may appear dull in comparison to applications for “ground-breaking” or “cutting-edge” innovation. It is imperative that this prejudice is removed so that wide-ranging, impactful IPC interventions can be researched, tested, costed, and optimally bundled.

In this study, we developed a list of sixteen IPC research priorities, supported by experts, including three urgent needs (Table 1). We encourage researchers, funding agencies, policymakers and relevant stakeholders to prioritise, fund, and research these identified gaps.

## Supplementary information

**Additional file 1.**

## Data Availability

The datasets used and analysed during the current study are available from the corresponding author on reasonable request.

## Abbreviations

AMR: Antimicrobial Resistance
ECDC: European Centre for Diseases Prevention and Control
EU-JAMRAI: European Joint Action on Antimicrobial Resistance and Healthcare-Associated Infections
ESCMID: European Society for Clinical Microbiology and Infectious Diseases
EUCIC: European Committee on Infection Control
HCAI: Healthcare Associated Infection
IMI: Innovative Medicines Initiative
IPC: Infection Prevention and Control
JPIAMR: Joint Programming initiative on Antimicrobial Resistance
LMIC: Low and Middle-Income Countries
OECD: Organisation for Economic Co-operation and Development
One Health-EJP: One Health European Joint Programme
SRIA: Strategic Research and Innovation Agenda
WHO: World Health Organisation
