# Supplementary figures and images for: Infection prevention and control research priorities: what do we need to combat healthcare-associated infections and antimicrobial resistance? Results of a narrative literature review and survey analysis
Source: Antimicrob Resist Infect Control. 2020 Aug 24;9:142. doi: 10.1186/s13756-020-00801-x (PMC7443818; doi:10.1186/s13756-020-00801-x)

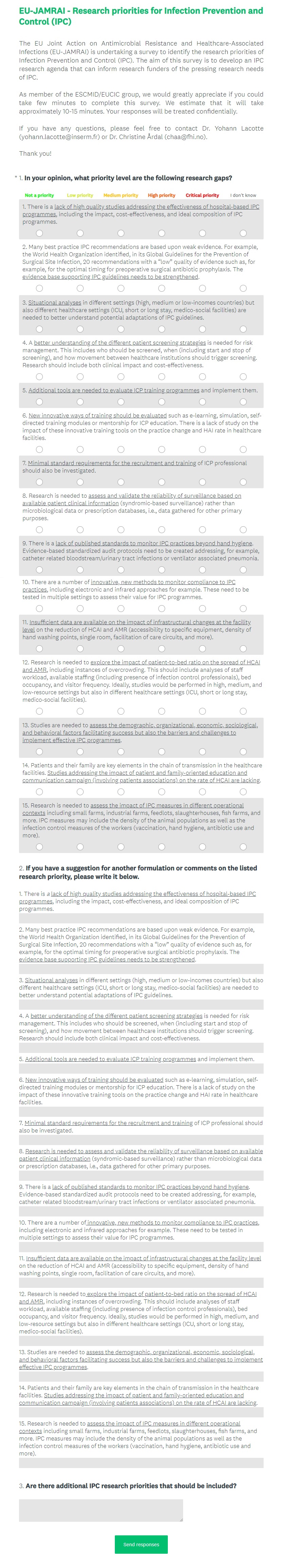

Supplement: Supplementary file 1 — Additional file 1. [file 13756_2020_801_MOESM1_ESM.jpg]
